# Nursing care for patients with pulmonary alveolar proteinosis undergoing whole-lung lavage therapy: Case report

**DOI:** 10.1097/MD.0000000000049958

**Published:** 2026-07-24

**Authors:** Liuyun Huang, Changjiao Chen, Mingying Zhao, Qiuxiu Qin

**Affiliations:** a Department of Geriatric Respiratory Medicine, The First Affiliated Hospital of Guangxi Medical University, Nanning, Guangxi, China.

**Keywords:** case report, nursing, perioperative, postoperative rehabilitation, pulmonary alveolar proteinosis, whole-lung lavage

## Abstract

**Rationale:**

Whole-lung lavage (WLL) is the definitive treatment for pulmonary alveolar proteinosis (PAP), yet it carries significant perioperative risks. Precision nursing is vital to optimize outcomes.

**Patient concerns:**

Four PAP patients presented with progressive dyspnea and severe anxiety regarding the complex surgical procedure and its uncertain prognosis.

**Diagnoses:**

The diagnosis was confirmed by the characteristic “paving stone” pattern on chest computed tomography and bronchoalveolar lavage fluid analysis.

**Interventions:**

A full-cycle precision nursing protocol was implemented, encompassing preoperative pulmonary prehabilitation and psychological support, intraoperative vital-sign monitoring with multidisciplinary collaboration, and postoperative airway management coupled with structured discharge follow-up.

**Outcomes:**

All patients completed WLL successfully with stable intraoperative physiology. No adverse events occurred. Postoperative respiratory function improved significantly, and therapeutic effects were sustained.

**Lessons:**

Full-cycle precision nursing ensures the safety and efficacy of WLL for PAP patients, optimizes perioperative management, prevents complications, and promotes patients’ long-term functional recovery. This standardized nursing model is feasible and worthy of clinical popularization.

## 1. Introduction

Pulmonary alveolar proteinosis (PAP) is a rare diffuse lung disease characterized by the accumulation of periodic acid–Schiff-positive phospholipoproteinaceous material within the alveolar spaces, with an estimated incidence of approximately 1 in 2 million.^[1,2]^ This pathological deposition severely compromises pulmonary ventilation and gas exchange, potentially leading to serious complications such as interstitial fibrosis, progressive dyspnea, and respiratory failure.^[2]^ At present, whole-lung lavage (WLL) remains the primary and most effective therapeutic intervention for PAP. The procedure involves the instillation of large volumes of sterile saline into the lung to mechanically clear lipoproteinaceous deposits, cytokines, and autoantibodies from the bronchoalveolar compartment, thereby alleviating clinical symptoms and slowing disease progression.^[3,4]^

From February 2022 to May 2023, 4 patients with PAP underwent WLL in the Department of Geriatric Respiratory Medicine, The First Affiliated Hospital of Guangxi Medical University. Because WLL is performed under general anesthesia and is highly invasive, perioperative complications – for example, lavage-fluid spillover, hypoxemia, pulmonary edema, and infection – are frequently reported.^[5,6]^ Consequently, a multidisciplinary team was convened before each procedure to devise an individualized surgical and nursing plan. Rigorous perioperative management was provided, and all 4 patients achieved symptomatic improvement and were discharged home. The findings before, during, and after lavage, along with follow-up results, are reported below.

## 2. Clinical data

### 2.1. General information

This group comprised 4 patients (2 males and 2 females) aged 42 to 56 years. The length of hospital stay ranged from 11 to 16 days. Other baseline characteristics are summarized in Table 1 and are described as occupation: patient 1 had worked in metal mold manufacturing for 21 years; the remaining 3 were farmers or held other occupations. Symptoms: all 4 patients presented with symptoms including chronic cough, sputum production, shortness of breath, and difficulty breathing; symptom duration ranged from 1 to 7 years. Smoking history: both male patients had a >20 pack-year history (≥30 cigarettes per day). Pulmonary function: 1 patient had severe restrictive ventilatory dysfunction, 2 had moderate restriction, and 1 had normal spirometry. Pathology: periodic acid–Schiff staining was positive in all 4 patients. Arterial blood gas analysis: all patients exhibited hypoxemia Chest computed tomography (CT): all scans showed characteristic crazy-paving or cobblestone patterns.

**Table 1:** General information of 4 patients with pulmonary alveolar proteinosis.

Patient	Age (yr)	Gender	Occupation	Hospital stay (d)	Reason for admission	Past medical history	Smoking status	Arterial blood gas analysis	PAS stain
1	47	Male	Farmer	14	Recurrent exertional dyspnea for >2 yr	None	40 cigarettes/day, 20 yr	Hypoxemia	+
2	42	Male	Metal mold manufacturing worker (21 yr)	16	Progressive worsening of cough and dyspnea for 3 mo	None	30 cigarettes/day, 21 yr	Hypoxemia	+
3	42	Female	Other	11	Recurrent cough and dyspnea for 7 yr	None	Nonsmoker	Hypoxemia	+
4	56	Female	Farmer	16	Cough and sputum production for >1 yr	None	Nonsmoker	Hypoxemia	+

Patient	Pulmonary function test			Lung biopsy			Chest CT findings		
1	Moderate restrictive ventilatory dysfunction			Interstitial changes with scant chronic inflammatory cell infiltration and carbon deposition			Bilateral patchy, flaky, and grid-like high-density shadows		
2	Severe restrictive ventilatory dysfunction			Uniform, diffuse accumulation of macrophages in the alveolar spaces			Bilateral large patchy ground-glass opacities, presenting with a “paving stone” pattern		
3	Moderate restrictive ventilatory dysfunction			Not performed			Bilateral multiple grid-like, nodular, and ground-glass opacities, showing a “paving stone” pattern		
4	Essentially normal pulmonary function			Chronic inflammation of bronchial mucosa and lung tissue			Bilateral multiple patchy and flaky ground-glass opacities, exhibiting a “paving stone” pattern		

CT = computed tomography, PAS = periodic acid–Schiff.

### 2.2. Treatment methods and outcomes

Patients were positioned supine and received total intravenous general anesthesia. Under fiber-optic bronchoscopic guidance, a left-sided double-lumen endotracheal tube was inserted; correct placement and complete lung separation were confirmed. While 1 lung was ventilated with 100% O_2_, the contralateral lung was lavaged.^[7,8]^ All patients underwent sequential WLL (left first, then right). Add 500 to 10,000 mL of 0.9% saline solution (heated to 37°C) in increments, and recover it under controlled negative pressure until the effluent changes from milky white to pale or completely clear.^[9]^ Representative changes in the color and clarity of the drainage fluid are shown in Figures S1 and S2, Supplemental Digital Content 1 and 2.

After completion of left-sided lavage, thorough bronchoscopic suctioning was performed. The patient was returned to the supine position and bilateral ventilation was initiated with positive end-expiratory pressure 5 cm H_2_O (0.49 kPa) and fraction of inspired oxygen 1.0. Oxygen saturation was maintained ≥94% and arterial blood-gas analysis confirmed PaCO_2_ ≈ 45 mm Hg.^[10,11]^ The identical protocol was then applied to the right lung.

Following the procedure, all 4 patients were transferred to the intensive care unit for respiratory and hemodynamic monitoring. Once extubated and stable, they were returned to the ward. All reported immediate relief of dyspnea and marked reduction in cough; cyanosis had resolved and hypoxemia had improved compared with prelavage levels. No atelectasis or pulmonary edema occurred. Chest CT performed 2 to 4 weeks later showed increased vascular markings and substantially reduced parenchymal attenuation. A comparison of lung CT results before and after lavage is shown in Figures S3A, B and S4A, B, Supplemental Digital Content 3 and 4.

## 3. Nursing

### 3.1. Preoperative care

The following items must be completed within 24 hours of admission: orientation: introduce the hospital environment, ward policies, the attending physician, and the charge nurse to the patient. Preoperative examinations and preparation: arrange and complete necessary tests, including pulmonary function tests, arterial blood gas analysis, blood specimen collection, and an electrocardiogram. Patients must fast for 8 hours and refrain from drinking for 4 hours before surgery.^[12^ Psychological care: patients and their families may experience anxiety or fear because of a lack of understanding about PAP and unsatisfactory results from previous drug therapies. Nurses should provide proactive psychological support by explaining the purpose, procedure, and potential benefits of the WLL, sharing successful case studies, and building the patient’s confidence to overcome the disease and cooperate actively with the treatment plan.^[13^ Preoperative training: preoperatively, teach patients effective coughing techniques, pursed-lip breathing, and aerobic exercise training. The goal is to enable patients to effectively cough up residual lavage fluid from the lungs, prevent alveolar adhesions and collapse, and improve lung capacity.^[14^ Final preoperative check: before the patient is transferred to the operating room, confirm that the surgical informed consent form has been signed and that the patient has changed into a hospital gown. Ensure that all necessary patient medications and items are handed over to the operating room nurse.

### 3.2. Intraoperative care

#### 3.2.1. Thermal preservation

The WLL procedure requires large-volume saline infusion and general anesthesia; prolonged operative time frequently overwhelms physiological thermoregulation and predisposes patients to hypothermia. Hypothermia impairs immune function and increases the incidence of intraoperative and postoperative complications.^[15]^ To prevent this, the team implemented the following measures: the operating room was maintained at 23°C to 25°C; warming blankets were activated 10 minutes before induction to preheat the table and linens; all infused and irrigating fluids were warmed to 37°C with fluid warmers.^[16]^ Rectal temperature was monitored continuously to keep body temperature between 36.5°C and 37.5°C. No intraoperative shivering was observed in any patient.

#### 3.2.2. Nursing collaboration

The nurse assisted the physician throughout the WLL procedure. After the anesthesiologist had performed double-lumen endotracheal intubation and initiated single-lung ventilation under general anesthesia, satisfactory lung separation was confirmed with a fiber-optic bronchoscope. The patient was turned to the left lateral decubitus position (head-up, foot-down) and the right lung ventilated with 100% oxygen. A 500 mL bag of 0.9% sodium chloride was hung from an IV pole at a height ≤50 cm above the bronchial limb. One end of the infusion line was connected to the lavage port of the double-lumen tube via a dedicated connector, and the opposite end was attached to a drainage bottle. Aliquots of 500 mL were instilled and left in the lung for 10 minutes; the line was then clamped. The patient was repositioned to the right lateral decubitus (head-down) position, the drainage channel was opened, and fluid was allowed to drain by gravity until flow ceased before the next cycle began. Chest percussion with a vibratory sputum-removal device was applied during both instillation and drainage to enhance protein evacuation. Lavage was continued until the effluent was clear.^[6]^ For detailed information on the irrigation process, see Table 2.

**Table 2:** Information on the WLL process for 4 PAP patients.

Patient	Operation duration	Total lavage fluid (mL)			Changes in the lavage fluid	Lowest arterial oxygen saturation (%)	pH	Highest PaCO_2_ (mm Hg)
		In	Out	Recovery rate (%)				
1	7 h	16,000	15,200	95.0	The lavage fluid gradually cleared from turbid to clear	70	7.107	64.1
2	8 h 50 min	19,000	18,450	97.1	The lavage fluid changed from milky to gradually clearer	92	7.264	45.5
3	6 h 40 min	20,000	19,600	98.0	The lavage fluid changed from milky to gradually clearer	94	7.320	39.0
4	6 h 20 min	20,000	19,000	95.0	The lavage fluid changed from milky to gradually clearer	91	7.162	43.0

PAP = pulmonary alveolar proteinosis, PaCO_2_ = partial pressure of arterial carbon dioxide, WLL = whole-lung lavage.

#### 3.2.3. Liquid management

Fluid management was a critical component of perioperative care. During WLL, the instillation of large volumes of normal saline into the lungs resulted in partial alveolar absorption. This process could damage pulmonary capillary walls, increase permeability, and predispose patients to volume overload and pulmonary edema. Therefore, invasive arterial blood pressure monitoring was established, and goal-directed fluid therapy was implemented to maintain balance.^[17]^ The intravenous infusion rate was controlled at 30 to 40 drops/minute, with strict recording of intake and output. The lavage infusion rate was set at 250 to 300 mL/min, with 500 mL per cycle. The intake-output balance was accurately recorded for each cycle. Lavage was immediately suspended if the cumulative negative balance (input exceeding output) exceeded 1000 mL. Following this, bronchoscopy was performed to verify endotracheal tube position and suction any excess pulmonary fluid. The total fluid recovery rate in this patient group ranged from 95.0% to 98.0%.

#### 3.2.4. Strengthen oxygen saturation monitoring

The patients’ vital signs, particularly blood oxygen saturation, were closely monitored throughout the procedure. During the right-lung lavage of patient 1, 2 episodes of oxygen desaturation occurred, with values fluctuating between 70% and 80%. Upon detection, the irrigation was immediately halted. Breath sounds were auscultated, the tidal volume was increased, and bronchoscopy was performed, which confirmed correct endotracheal tube placement without fluid leakage in the ventilated lung. Excess fluid was suctioned from the lavaged lung, and an intravenous bolus of 20 mg furosemide was administered. Arterial blood gas and serum electrolyte tests were promptly conducted. On the basis of the results, the blood volume was replenished, after which the patient’s oxygen saturation rose to above 95%. The other 3 patients did not experience any significant or persistent decreases in oxygen saturation during the lavage.

### 3.3. Postoperative care

#### 3.3.1. Airway management

All patients in this group were intubated with a single-lumen cuffed endotracheal tube for postoperative ventilation and were immediately transferred to the intensive care unit for monitoring and treatment. The head of the bed was elevated to 30° to 40°, and nebulizer therapy was administered to facilitate sputum clearance. Patients were regularly turned and received back percussion. Airway suctioning was performed as needed to maintain patency. Sputum was observed for the presence of pink, frothy secretions. Once all monitoring parameters met the weaning criteria, the endotracheal tube was removed, and noninvasive ventilator support was initiated. Oxygen flow was initially set at 5 L/min and adjusted according to the patient’s condition. During this period, arterial oxygen saturation was maintained at approximately 95%.

#### 3.3.2. Complication monitoring

1. Hypoxemia: The most common complication of WLL is hypoxemia, which frequently occurs during the drainage of lavage fluid. A reduction in airway pressure can increase perfusion to the lavaged lung, leading to an abnormal ventilation-perfusion ratio.^[18]^ Therefore, targeted nursing interventions were implemented. Postoperatively, patients received mechanical ventilation via an endotracheal tube. Interventions such as regular turning, back percussion, and suctioning were performed to facilitate the drainage of residual lavage fluid. Arterial blood gas analysis was monitored regularly, and the inspired oxygen concentration was adjusted accordingly. After extubation, noninvasive ventilation was initiated with an oxygen flow rate of 5 L/min, which was further titrated based on arterial blood gas analysis results. These measures successfully maintained arterial oxygen saturation above 95% throughout the recovery period.
2. Acute pulmonary edema: The instillation of large volumes of normal saline during WLL washes out alveolar deposits but also removes pulmonary surfactant. In addition, partial alveolar absorption of the saline can damage pulmonary capillary walls and increase their permeability, thereby elevating the risk of acute pulmonary edema.^[5]^ To mitigate this risk, the following measures were implemented postoperatively: administration of prescribed furosemide and methylprednisolone, 24-hour electrocardiogram monitoring, and strict recording of fluid balance. Total fluid volume and infusion rates were rigorously controlled. None of the patients in this group developed signs of pulmonary edema, such as worsening dyspnea, respiratory distress, cyanosis, diaphoresis, restlessness, or pink frothy sputum during the observation period.
3. Pulmonary infection: In PAP patients, compromised alveolar macrophage function impairs respiratory defense mechanisms. Furthermore, the accumulation of proteinaceous material in the alveoli provides a medium for bacterial growth, increasing the risk of secondary infection.^[19]^ In this case series, patient 4 developed a fever (37.5–38.3°C) after WLL. This was managed with anti-infective therapy (ceftizoxime) and physical cooling. The other 3 patients remained afebrile. In addition, all patients were instructed in diaphragmatic breathing to improve gas exchange efficiency and reduce the work of breathing, as well as effective coughing techniques to help expel residual lavage fluid.
4. Electrolyte imbalance: The instillation of large volumes of normal saline during WLL, combined with the prophylactic use of diuretics to prevent pulmonary edema, predisposes patients to electrolyte disturbances.^[19]^ Accordingly, all patients underwent postoperative 24-hour electrocardiogram monitoring, with close observation of heart rate and rhythm, supplemented by arterial blood gas analysis. In one instance (patient 2), blood gas analysis following WLL revealed hypokalemia, with a serum potassium level of 2.4 mmol/L. This condition was corrected with oral potassium supplementation and a potassium-rich diet, which raised the serum level to 3.7 mmol/L. The remaining 3 patients maintained normal potassium levels.

#### 3.3.3. Pulmonary rehabilitation nursing

The charge nurse assessed secretion status and instructed patients to use a vibrating secretion clearance device twice daily for 10 to 15 minutes to loosen secretions. Position changes were then used to further facilitate sputum movement. Patients were encouraged to perform effective coughing and diaphragmatic breathing. To evaluate the recovery of pulmonary function, oxygen supplementation was temporarily discontinued for 15 minutes. In this group, patients maintained oxygen saturation levels of 95% to 98% with low-flow nasal cannula oxygen therapy and 90% to 95% without it. Before discharge, patients’ mastery of pulmonary rehabilitation exercises and their overall readiness for discharge were assessed.

### 3.4. Follow-up care

After discharge, patients’ self-management outcomes were monitored through automated electronic messages and telephone follow-ups. The assessments covered: whether workplace changes or protective measures had been implemented; the resolution of symptoms such as cough, sputum, and dyspnea as an indicator of recovery; and adherence to health guidance, including avoiding cold exposure to prevent infections, engaging in appropriate exercise, maintaining a work-rest balance, consuming a balanced diet, using protective measures to avoid inhaling harmful dust or gases, and attending regular outpatient reviews. During the follow-up period, patients reported an absence of chest tightness, dyspnea, and cough, alongside a gradual improvement in tolerance for light physical activity. Pulmonary CT scans conducted 2 to 4 weeks postdischarge showed improvement compared with preoperative baseline scans, and patients’ psychological status remained stable. Notably, patient 2 ceased employment in metal mold manufacturing, and both male patients successfully quit smoking. The patients in this group were followed for 18 months to 3 years.

## 4. Summary

PAP is a rare clinical disorder for which WLL is a safe and effective treatment. It alleviates clinical symptoms and delays the progression of pulmonary fibrosis. The patients in this series presented with hypoxemia and diminished pulmonary reserve, which are risk factors for complications during WLL. Therefore, meticulous, comprehensive, and individualized perioperative nursing care was essential to ensure the procedure was well-tolerated and to effectively reduce the incidence of complications. However, as this study was a single-center, retrospective analysis with a limited sample size, it is susceptible to selection bias and incomplete documentation of nursing interventions. Future efforts will aim to accumulate clinical experience to improve the management of adverse reactions related to WLL in patients with PAP.

## Acknowledgments

We sincerely thank each patient and their family members for their willingness to provide case information for our research. Additionally, we also thank all the authors who contributed to this article.

## Author contributions

**Conceptualization:** Liuyun Huang, Changjiao Chen.

**Data curation:** Liuyun Huang, Mingying Zhao, Qiuxiu Qin.

**Validation:** Liuyun Huang.

**Project administration:** Changjiao Chen.

**Writing – original draft:** Liuyun Huang, Changjiao Chen.

**Writing – review & editing:** Changjiao Chen.








