# Supplementary figures and images for: Nursing care for patients with pulmonary alveolar proteinosis undergoing whole-lung lavage therapy: Case report
Source: Medicine (Baltimore). 2026 Jul 24;105(30):e49958. doi: 10.1097/MD.0000000000049958 (PMC13406321; doi:10.1097/MD.0000000000049958)

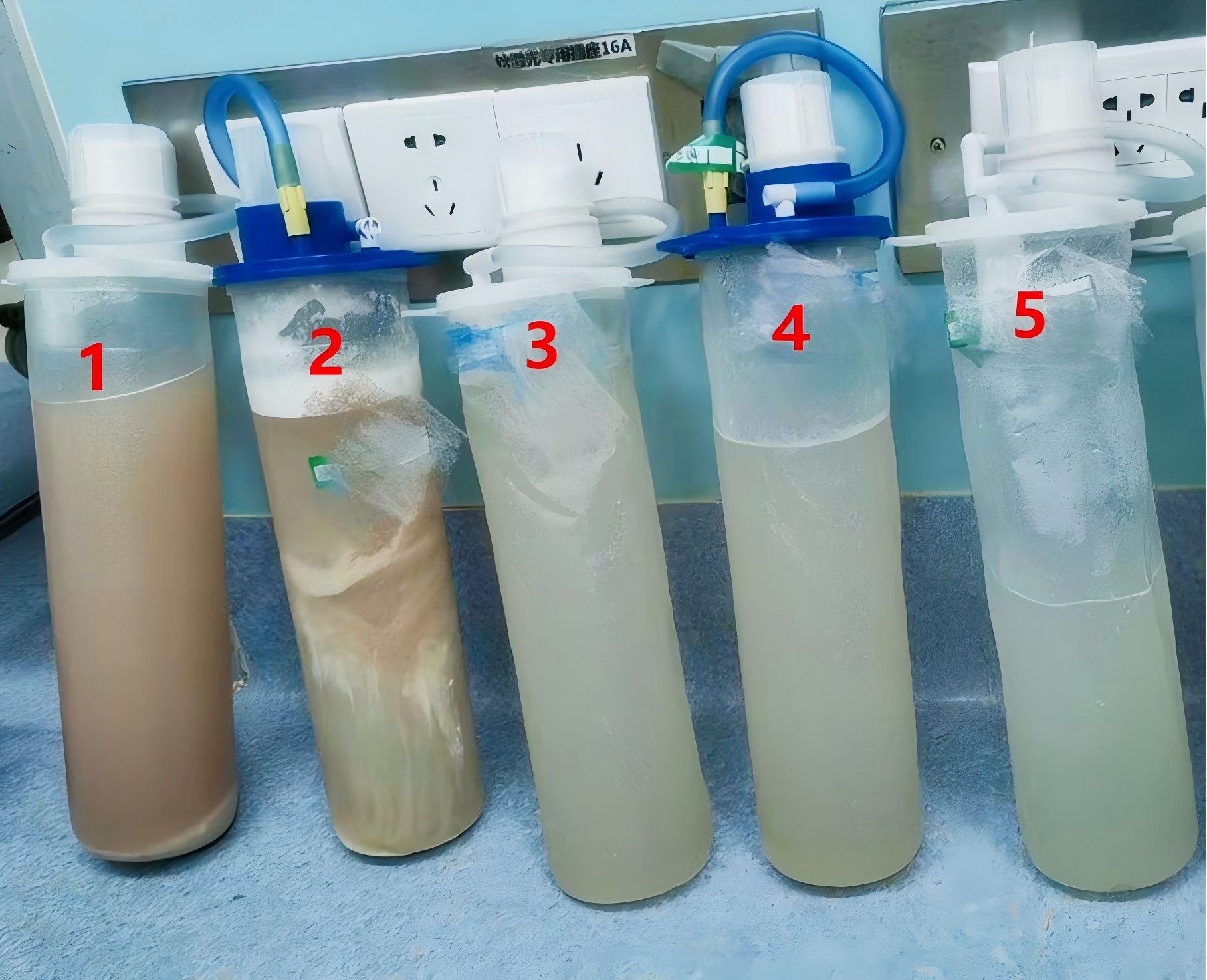

Supplement: Supplementary file 1 [file medi-105-e49958-s001.jpg]

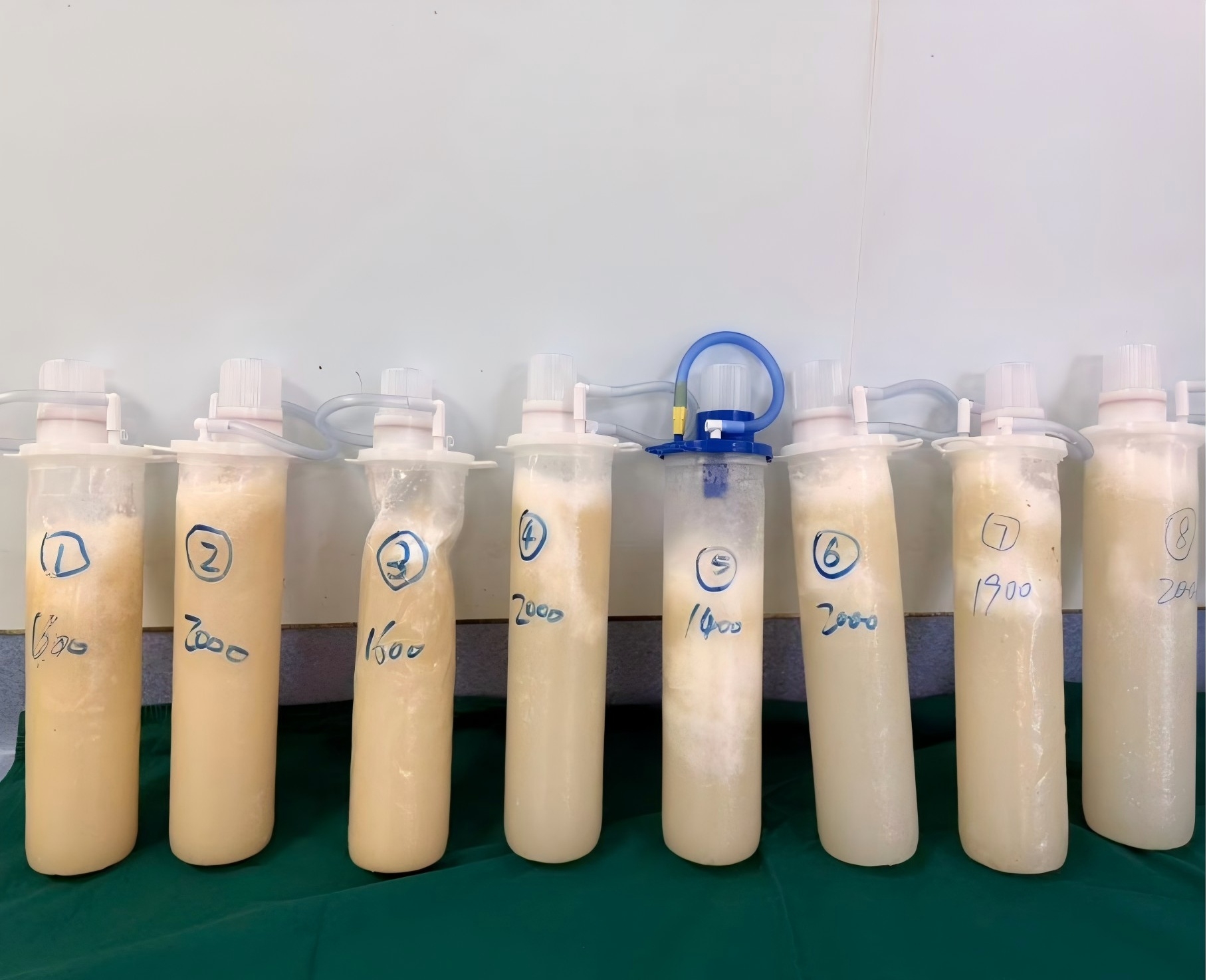

Supplement: Supplementary file 2 [file medi-105-e49958-s002.jpg]

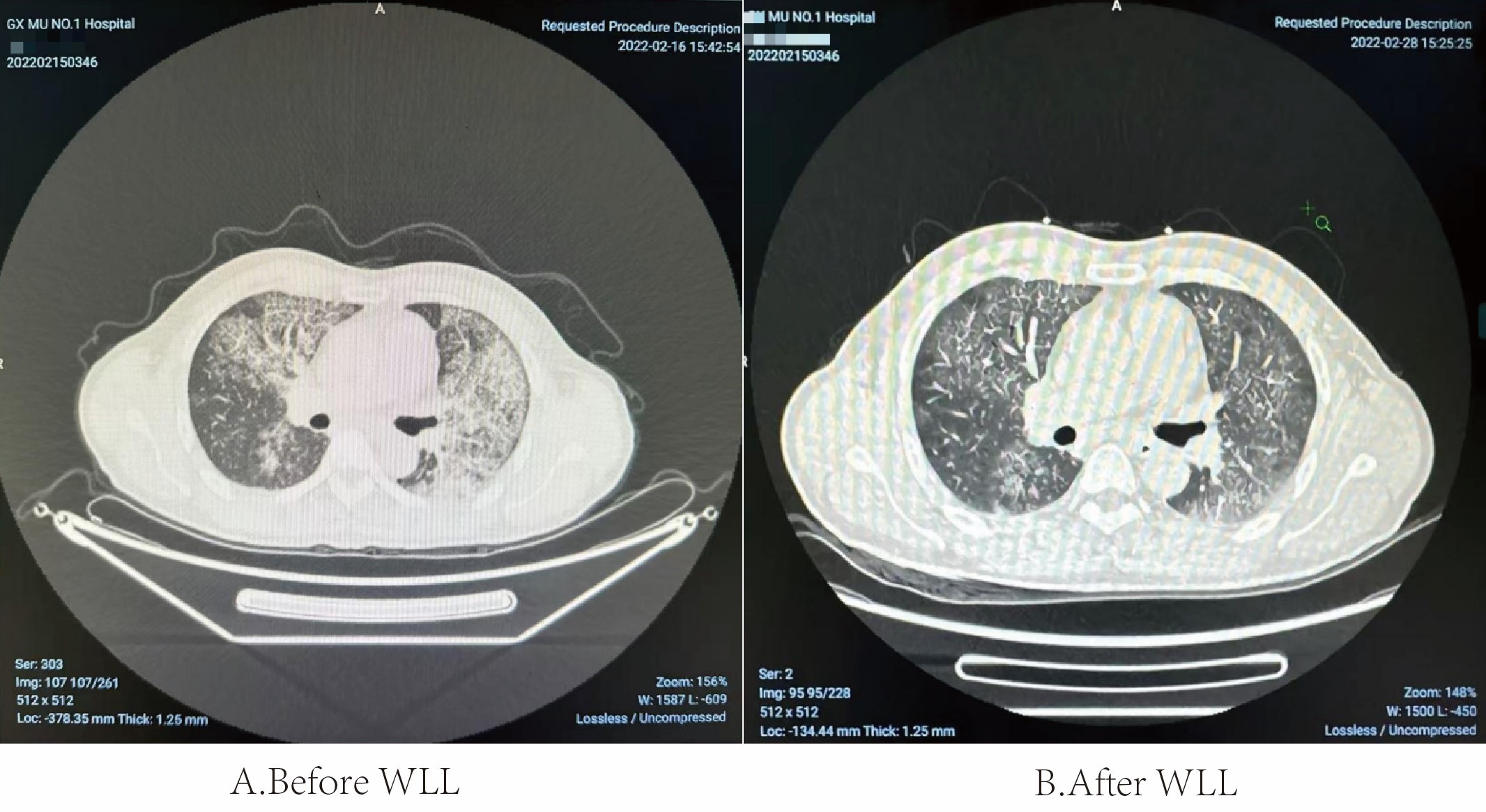

Supplement: Supplementary file 3 [file medi-105-e49958-s003.jpg]

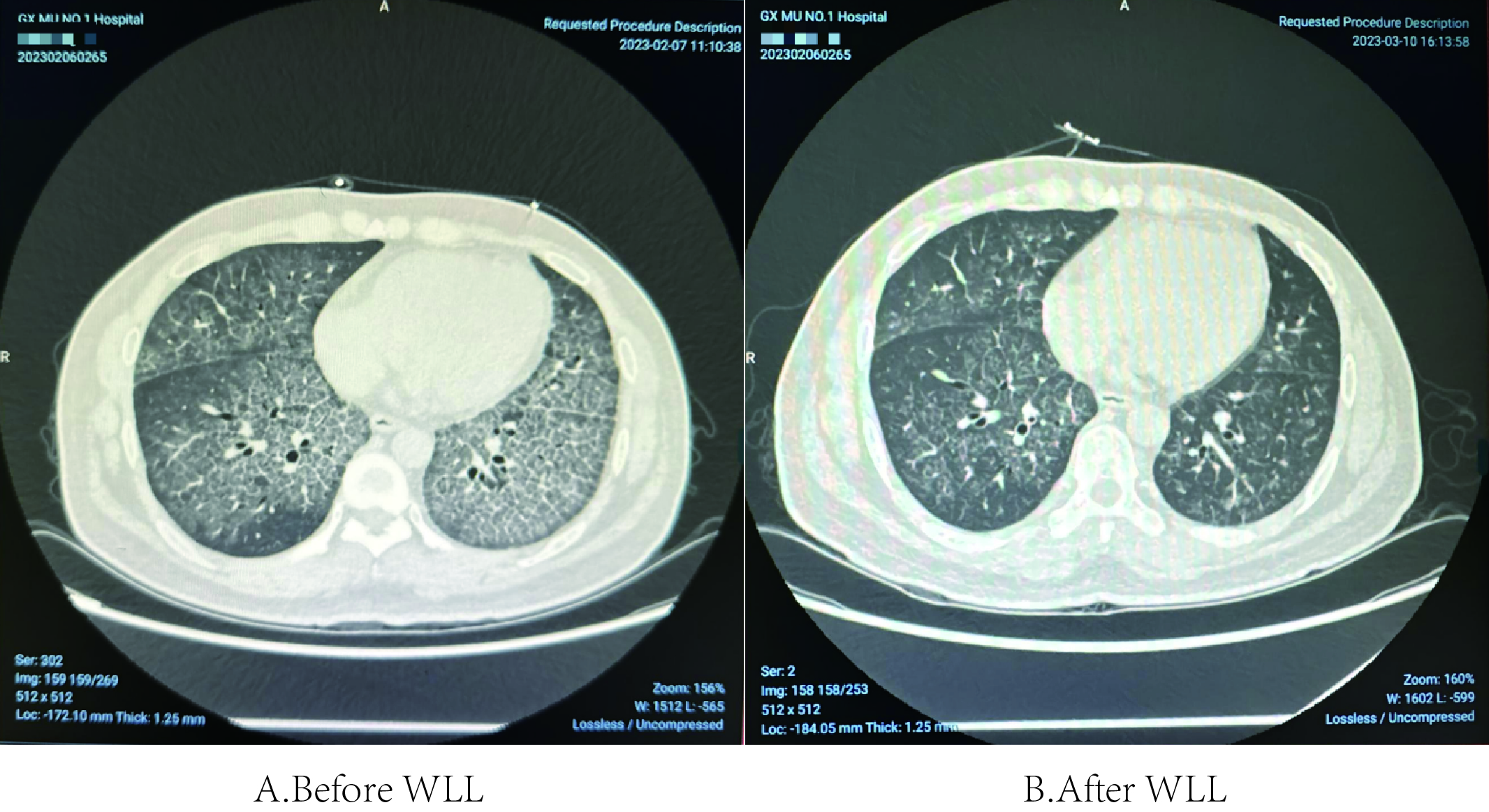

Supplement: Supplementary file 4 [file medi-105-e49958-s004.jpg]
